# I Am a Compassionate Conservation Welfare Scientist: Considering the Theoretical and Practical Differences Between Compassionate Conservation and Conservation Welfare

**DOI:** 10.3390/ani10020257

**Published:** 2020-02-06

**Authors:** Ngaio J. Beausoleil

**Affiliations:** Animal Welfare Science and Bioethics Centre, School of Veterinary Science, Massey University, Private Bag 11-222, Palmerston North 4410, New Zealand; N.J.Beausoleil@massey.ac.nz

**Keywords:** conservation, animal welfare science, compassionate conservation, compassion, wildlife, ethics

## Abstract

**Simple Summary:**

The well-being of individual wild animals is threatened in many ways, including by activities aiming to conserve species, ecosystems and biodiversity, i.e., conservation activities. Scientists working in two related disciplines, Compassionate Conservation and Conservation Welfare, are attentive to the well-being of individual wild animals. The purpose of this essay is to highlight the commonalities between these disciplines and to consider key differences, in order to stimulate discussion among interested parties and use our collective expertise and energy to best effect. An emerging scenario, the use of genetic technologies for control of introduced animals, is used to explore the ways each discipline might respond to novel conservation-related threats to wild animal well-being.

**Abstract:**

Compassionate Conservation and Conservation Welfare are two disciplines whose practitioners advocate consideration of individual wild animals within conservation practice and policy. However, they are not, as is sometimes suggested, the same. Compassionate Conservation and Conservation Welfare are based on different underpinning ethics, which sometimes leads to conflicting views about the kinds of conservation activities and decisions that are acceptable. Key differences between the disciplines appear to relate to their views about which wild animals can experience harms, the kinds of harms they can experience and how we can know about and confidently evidence those harms. Conservation Welfare scientists seek to engage with conservation scientists with the aim of facilitating ongoing incremental improvements in all aspects of conservation, i.e., minimizing harms to animals. In contrast, it is currently unclear how the tenets of Compassionate Conservation can be used to guide decision-making in complex or novel situations. Thus, Conservation Welfare may offer modern conservationists a more palatable approach to integrating evidence-based consideration of individual sentient animals into conservation practice and policy.

## 1. Introduction

I am an animal welfare scientist—my aim is to use scientific methods to explore the experiences of sentient animals and the ways in which our human systems and behaviours influence those experiences and thus the animals’ lives. The knowledge I generate in this way is sometimes used to alter human systems and behaviour in an attempt to uphold an implicit and sometimes explicit ethic: that it is wrong to cause unnecessary suffering or harm to animals (e.g., the New Zealand Animal Welfare Act [1] makes it unlawful to cause animals “unreasonable or unnecessary pain or distress”). 

Some of my work focuses on wild animal welfare, a sub-discipline referred to as ‘Conservation Welfare’ because many of the harms that can be experienced by wild animals are associated with our attempts to redress what we have done to the planet and its occupants, both human and otherwise [2]. These conservation activities include moving, containing/confining, excluding and killing large numbers of animals [3,4,5,6].

Concern for the well-being of individual wild animals is widespread among the public and conservationists. For example, three-quarters of respondents declaring an interest in conservation (both conservation professionals and members of the American public) agreed that we are obliged to treat wild animals with concern for their welfare [7]. However, views vary on the character and extent of our obligations to individual wild animals and how those obligations interact with responsibilities to protect other aspects of ‘nature’ (e.g., populations, species, ecosystems, biodiversity) [3,4,8,9,10,11,12,13,14,15]. It is beyond the scope of this essay to outline the numerous environmental and animal ethics but two disciplines, Compassionate Conservation and Conservation Welfare, specifically focus attention on the well-being of individual wild animals [2,3,5,16,17].

Common positions about which wild animals are morally considerable are as follows: only animals that have instrumental value to humans should be considered, a position which is effectively a proxy for the interests of humans; only sentient animals have intrinsic value (valued for their own sake) and should be considered, i.e., those that have a capacity to take an interest in their own lives; all animals should be included and their interests should be considered equally, including with those of humans [4,11,12,18]. Both Compassionate Conservation and Conservation Welfare ascribe intrinsic value to some individual wild animals and endorse our moral obligation to consider their well-being, i.e., some animals have some degree of moral standing. However, what those obligations are and how they may be fulfilled appears to differ between the disciplines. Fundamental differences in philosophy sometimes lead to conflicting views about the kinds of conservation activities and decisions that are ethically acceptable [19]; thus, contrarily to what has been implied [9,20,21,22], the two disciplines are not the same.

Highlighting differences in our underlying ethics and their practical implications can be useful if it helps us understand each other. However, focusing on differences may create siloed attempts to address the same problems or even undermine collegial or public confidence in the work of each discipline [6]. This can make us less effective overall and less open to participating in collective efforts on behalf of wild animals. In contrast, exploring our commonalities can facilitate constructive dialogue [23,24] and enable each party to reinforce the other’s actions taken to support the well-being of wild animals. 

In this spirit, this essay explores ways in which those subscribing to Compassionate Conservation and those aligning with Conservation Welfare are similar in their thinking and action. The main aim is to stimulate discussion among those having concerns about the well-being of both individual wild animals and the collectives and ecosystems to which they belong. In addition, I seek clarification for myself (and for interested others) of the salient differences between the two disciplines. To illustrate, I speculate on the ways in which Compassionate Conservation and Conservation Welfare scientists might approach a novel conservation activity: gene editing for the purposes of eradicating introduced species. Finally, I comment on the suitability of each approach for integrating consideration of individual animal welfare into modern conservation practice and policy. 

To set the scene, I will first briefly characterize the key features of each discipline, including the commonly referenced ethical bases. This is not a straightforward task, particularly as Compassionate Conservation is an evolving discipline and not fully articulated in the literature [7,25,26] and there appears to be some confusion about the ethical basis of its four tenets and their practical application [6,22,26,27,28].

## 2. Compassionate Conservation

Compassionate Conservation is proposed as an interdisciplinary approach to integrating conservation and animal protection ethics to achieve conservation outcomes while minimizing harms to the welfare of individual animals [29]. Implicit in this proposal is the view that individual wild animals have intrinsic value and thus moral standing, that is we are obliged to have concern for their welfare and treat them in a just manner [11,16]. When explored in more detail, Compassionate Conservation seems to represent a pluralist ethical approach which prioritizes the well-being of individual wild animals. Features of a virtue ethic, a deontological ethic and perhaps consequentialism are evident in various descriptions of the approach published over the last decade or so. 

Central to Compassionate Conservation is the notion that a person of good character shows compassion [5]; a virtuous person is one who is moved by the suffering or distress of others, in this case wild animals, and who feels a desire to relieve that suffering [8,16]. Virtue ethics focus on the intention of the moral actor and their character to decide which actions are morally acceptable and are not concerned with the outcomes of those actions, e.g., whether the actions actually relieve suffering or not [30]. 

Some [22,28] contend that Compassionate Conservation also incorporates an animal rights position (compare to [16]). According to deontological ethics, of which animal rights is an example, the rightness of an action is determined by its compatibility with a set of prescribed rules or duties [11,30]. Evidence of a rights position in Compassionate Conservation is cited [22,28] as its rejection of utilitarianism (i.e., Animal Welfare, see below) as justifying mass suffering of wild animals [16] as well as the fundamental ‘wrongness’ of killing [5,26,31], which seems to align with the ‘right not to be killed’ and the duty of moral agents to respect that right [11,32].

Finally, there is some informal evidence that some Compassionate Conservationists do consider the consequences of actions, in that they advocate seeking and applying conservation methods that have better consequences (i.e., less welfare impact) for wild animals (see *First, do no harm* below) [29,33]. In addition, the success of compassionate conservation activities for safeguarding other aspects of nature is considered [5,31]. Four tenets are articulated to guide those aiming to achieve compassion in conservation; the ways in which these tenets relate to the practice of Conservation Welfare are considered in detail in later sections.

## 3. Conservation Welfare

Conservation Welfare reflects the relatively recent application of the animal welfare ethic to consider the effects of conservation activities on free-living wild animals. Much of the development in this sub-discipline has been driven by concerns about the effects of methods used to control invasive or otherwise unwanted animals [17,34,35,36,37,38,39,40] though the potential for conservation activities to impact both negatively and positively on animal welfare has been explored more broadly [10,41,42,43,44,45,46,47,48,49].

Animal welfare is a consequentialist ethic generally following Singer’s utilitarianism, according to which the right action is the one that results in the greatest good for the greatest number [50]. In accordance, it is acceptable to cause some harm to some animals if those harms are outweighed by the anticipated benefits accrued by all ‘considerable’ parties, i.e., those having significant interest in the outcomes [2,28,50]. Of course, in reality, this kind of calculation is complex and difficult (if not impossible) to make, and there is diverse opinion about which parties are ‘considerable’ and whose interests should be given greatest weight in the calculation. Thus, the principle is usually applied in practice by trying to minimize harms and maximize benefits [35,51], and in order to even attempt this, the harms and benefits must be understood and somehow quantified [8,17,28,52]. Sometimes developing this understanding causes harm to some animals, e.g., during wildlife research [44,47,53,54].

Animal welfare is also predicated on the central notion that is it wrong for people to cause ‘unnecessary’ suffering or harm to those animals capable of experiencing suffering or harm [50,55] and this is a key feature of the legal protection of animals in many countries [56]. As in Compassionate Conservation, this concept implies the intrinsic value of individual animals and thus, some degree of moral obligation towards them [50]. However, the animal welfare ethic arose largely from considerations of the treatment of domestic animals kept for purposes such as food production and scientific research, and thus, inherently accepts some uses of animals for human purposes even if harms are caused [3,50,57]. According to the animal welfare ethic, there is no ethical problem with killing animals, even if it is done unnecessarily, because killing itself does not harm them (as long as it is done without causing suffering; see *First do no harm* below) [50]. Our moral obligation is to spare them suffering that is unnecessary, and a key ethical conundrum is ‘what use of animals, and what suffering, is necessary?’.

Closely associated with the animal welfare ethic is the discipline of animal welfare science [51] which applies scientific methods to inform an understanding of what harms (and benefits) animals might be able to experience, in which circumstances they might experience them, how particular harms compare to the harms resulting from alternative actions, and how successful any attempts to mitigate harms have been [58,59,60]. This information can then be used to inform decision-making about which actions are morally and ethically permissible. In theory, animal welfare science itself is ethically neutral; the information generated could equally be used to support an argument that no human uses of animals are acceptable because they all cause ‘unnecessary’ harm as it could be used to permit animal use [50]. It does not address the issue of the necessity of harms because this is an ethical, not a scientific question. However, many of the research questions explored are chosen because of a desire by animal welfare scientists to avoid or minimize animal suffering (i.e., the science is predicated on the animal welfare ethic) [51].

As Conservation Welfare simply represents a newish application of an existing ethic and associated scientific methods, some may argue that using the term ‘Animal Welfare’ in the context of conservation is sufficient. However, I assert that there is value in specifically labelling this application of welfare thinking and science applied to wild animals for two main reasons. First, it is useful to bring the need to consider individual wild animal welfare to the attention of conservation biologists and policy-makers. Second, there is a need to clearly differentiate this approach to considering wild animal welfare from other approaches, including Compassionate Conservation, due to important differences that will be discussed below.

## 4. The Four Tenets of Compassionate Conservation

Compassionate Conservationists espouse the view that although it is critical to undertake activities that safeguard biodiversity, these activities can and should be done in ways that manifest compassion towards individual wild animals [5,16,20,21,31,61]. More specifically, the enactment of compassion in conservation should be guided by the four Compassionate Conservation principles: (1) First, do no harm; (2) Individuals matter; (3) Inclusivity; (4) Peaceful co-existence. Here, I explore each principle in turn, beginning with the second, and consider the ways in which the practice of Conservation Welfare is similar and different. 

### 4.1. Individuals Matter

It is my view, as an animal welfare scientist, that only individual animals matter when considering welfare. In this regard, collectives, such as populations and species, matter because they are made up of numerous individuals [2]; this is apparently consistent with the Compassionate Conservation definition of compassion (Wallach et al. 2018 p1262) [5]. Nevertheless, collectives of animals also matter because they are required to ensure the welfare of individuals within them. For example, a critical mass of animals or normal social structure may be needed to ensure safety from predators or for breeding success, or to provide sufficient biodiversity to avoid pathological conditions associated with inbreeding, such as unusual disease susceptibility [62,63,64]. Thus, from a Conservation Welfare perspective, collective-level conservation goals can be important because they contribute to protecting the welfare of individual animals. 

In practical terms, one way to highlight the importance of individual wild animals in conservation planning and practice is to use a characterization of animal welfare that centres on the animal’s mental/affective experiences [2]. Focusing attention on how animals *feel* about their environment and what happens to them in it is one way to encourage moral consideration of individuals by creating an obligation to minimize or prevent their suffering, thus encouraging compassion in conservation.

With regard to the principle of ‘Individuals matter’, there may be a key difference between the positions adopted within Conservation Welfare and Compassionate Conservation; those working under the banner of Conservation Welfare would usually afford that consideration only to individuals of species capable of taking an interest in their own experiences and lives, i.e., sentient animals [65,66]. Inevitably, which individuals get to be in this ‘sentience club’ reflects our own subjective standards and our limited understanding at any point in time. In New Zealand legislation, for example, sentience is currently attributed only to vertebrates and the cephalopod and decapod crustacean invertebrates [1; Section 2: Definition of ‘animal’]. 

However, given our uncertainty about which animals possess a capacity for sentience and thus our growing discomfort about excluding species that might meet the ‘club membership criteria’ [67,68], do Compassionate Conservationists give all animals the benefit of the doubt? This is unclear. On the one hand, Wallach et al. [5] note that a virtuous person (according to Compassionate Conservation) will carefully attend to the capacity of others to experience both joy and pain; this sounds suspiciously like the definition of sentience. Earlier papers appear to apply a similar sentience threshold [16,25]. On the other hand, the counter argument made in Wallach et al.’s 2018 paper under *Inclusivity,* that individuals of *all* wildlife species be considered, regardless of their capacity for sentience, requires clarification, given its significant implications [6]. Considering the interests of individuals of *all* animal species, including parasitic worms, mammals, sponges, reptiles and arthropods, would make conservation decision-making considerably more challenging. 

### 4.2. Inclusivity

This third Compassionate Conservation tenet asserts that compassion should not be limited to those wild animals that have instrumental value for humans. Instrumental values can be, and sometimes are, successfully used as levers to achieve consideration of individual wild animals in conservation. This is a strategy commonly applied by animal welfare scientists in the contexts of both domestic and wild animals. We often use concurrent improvements in domestic animals’ instrumental value (e.g., improvements in productivity or ease of handling) to leverage improvements in their welfare [69,70]. Similarly, in Conservation Welfare, we aim to demonstrate that considering individual wild animal welfare also serves important conservation goals such as improving the survival of individuals of valued species [8,46,71,72], and this is apparently consistent with the practices in Compassionate Conservation (Table 2 in Wallach et al. 2018 and explicitly on p 1261) [5]. 

But, in contrast to some criticisms levelled at animal welfare scientists (e.g., that they use science to facilitate animal abuses [19,73]), I would argue that most do what they do because they believe that (at least some) animals have intrinsic value and that their welfare should be safeguarded because it is the ‘right thing to do’. In fact, like those who have founded the Compassionate Conservation movement, many animal welfare scientists have been drawn to our discipline because of a sense of injustice regarding the ways in which humans interact with other animals. We do, of course, generally accept that animals will continue to be used for human purposes, and this is apparently a key difference between Compassionate Conservation and Conservation Welfare. But this does not preclude our simultaneously held beliefs about the intrinsic value of sentient animals and the importance of compassion towards them.

Thus, only leveraging instrumental value to ensure consideration of individual wild animals is likely to be unsatisfying for those working in both disciplines. As Wallach et al. [5] point out, instrumental values change and some wild animals are not perceived to have any such value to humans. In these cases, individual animals are unlikely to inspire compassionate treatment, and in fact are sometimes explicitly exempted from such treatment to facilitate achieving other goals [2]. To illustrate, rarity or nativism could be construed as instrumental values, either because they are features fundamentally attractive in some worldviews [18] or because they confer more tangible benefits, e.g., economic value of native and/or rare animals for tourism [74]. But both rarity and nativism, and thus the value ascribed to certain animals, are non-fixed. For instance, once conservation action successfully recovers a population of previously rare animals, the perceived value of those animals may be reduced. This is sometimes coupled with a growing inconvenience to humans of having larger populations of these animals around human habitations (e.g., restored Kaka (*Nestor meridionalis*) populations in Wellington, New Zealand [75]).

For other animals, once-appreciated uses (e.g., for biocontrol or a commodity market such as the brushtail possum (*Trichosurus vulpecula*) fur trade in New Zealand) are no longer valued. For others still, many perceive no instrumental value, e.g., commensal rats and mice. Applying the Compassionate Conservation principle of inclusivity aims to protect the interests of individual wild animals regardless of their origin, current population size or value to humans. The problem is that the impacts of these animals on other resources valued by humans (e.g., agricultural crops) often means that there is little appetite to extend compassion to them – see *Peaceful Coexistence* below. Here, Conservation Welfare proponents take an intermediate position (and have been criticized by both Compassionate Conservationists and ‘traditional’ conservationists [19,47]), i.e., they acknowledge the desire of many to ‘manage’ such animals, but advocate doing so in ways that minimize the harm inflicted as well as developing less harmful methods [17,35]. While inadequate for many Compassionate Conservation proponents, this more moderate ‘incremental improvement’ approach may gain more traction with traditional conservationists [6,27,28,76] and thus generate large scale change more quickly. My own aim, and that of many other conservation welfare scientists, is to contribute to the development of new conservation paradigms that give greater weight to animal welfare alongside factors such as efficacy and cost when making decisions [17,39,41,46,52,77,78]. This may mean that some conservation activities have to be modified or that some activities cannot be undertaken until less harmful methods are developed [2].

### 4.3. Peaceful Coexistence

The fourth Compassionate Conservation tenet of Peaceful Coexistence urges us to always seek opportunities to resolve human-animal conflict in ways that do not harm the animals. In particular, we are encouraged to modify our own behaviour and practices to reduce potential conflict (e.g., managing waste), to rigorously explore the actual significance of the animals’ impacts (both positive and negative) and to find non-harmful ways to circumvent any impacts they have that are significantly negative. Wallach and colleagues give some inspiring examples of success in Compassionate Conservation [5]. Conservation Welfare scientists likewise recommend these actions as a first and critical step in systematic approaches to managing such conflicts [17,35]. 

However, peaceful coexistence becomes more challenging when the human behaviour leading to conflict is historical (e.g., animal introductions or range reduction), when the impacts are perceived by many to be significantly detrimental to people or other animals, or when non-harmful ways of avoiding those impacts are not currently available [8,26]. This is particularly challenging when achieving peaceful coexistence means accepting ‘novel ecosystems’, i.e., those that include ‘undesirable’ types or numbers of wild plants or animals [18,79]. The New Zealand situation exemplifies this challenge; the introduction, by European humans, of both wild and domestic animals has had profound effects on ‘pre-colonial ecosystems’ [80]. The perceived value of retaining or restoring past ecosystems is one of the bedrocks of modern conservation (e.g., Invasion and Restorations Biologies; [18,81]) and will be difficult to change, especially whilst this value is still strongly reinforced [82] (however, compare to [83,84]. 

When conflict between wildlife and humans cannot be avoided, Compassionate Conservationists advocate the use of minimally invasive and non-lethal methods to achieve peaceful co-existence. These include excluding or deterring wild animals from valued resources or areas using physical and visual barriers or guarding dogs, relocating ‘problem’ individuals and applying fertility control or encouraging natural predation to keep populations in check [5,31]. But the assumptions that such methods are effective in achieving other conservation goals (e.g., preserving populations or biodiversity) or that they minimize harm to the target animals are not always supported [22,27,85,86,87,88]. It is currently unclear how complex dilemmas between lethal control of unwanted animals to protect other animals and resources and existing peaceably without causing harm can be resolved following Compassionate Conservation [26]. 

From a Conservation Welfare perspective, peaceful coexistence is a desirable objective where it is possible and does not threaten the ‘overall good’, which is usually taken to mean that it does not preclude protection of other valued aspects of nature. When peaceful coexistence is not possible or desirable, Conservation Welfarists advocate the use of methods and systems that minimize harms to animals involved in conflicts. In the case of control of unwanted animals, this necessitates explicitly affording *all* sentient animals protection from intentional and ‘unnecessary’ harms [8], e.g., removing existing loopholes in laws that exempt ‘pests’ from such protection of their welfare [2,89,90]. Because of such exemptions, the claim made by Hayward et al. [6] that “methods used by professionals to kill animals for conservation purposes will almost always be more humane…than the methods used by animals to kill each other” is unlikely to be true, at least in the New Zealand context where much of the pest control undertaken involves poisons that cause intense and prolonged negative welfare impacts before death [91]. To promote the ‘greatest good’ also requires provision of clear evidence of the ways in which purported benefits will be realized [16,17,35,82], both in the course of approvals for ‘routine’ conservation activities and for wildlife research [44]. As an example, this means providing compelling information that control programmes will achieve outcomes that align with key values, e.g., Nature Rich/Biodiverse versus Predator Free [17,82].

Within the Conservation Welfare paradigm, progressing more peaceful coexistence also involves: prioritizing research that aims to find methods with fewer welfare impacts, including strategic funding of such research [34,92,93]; and systematically exploring the relative harms associated with existing approaches to facilitate application of those that cause least harm [40,87,91]. Importantly, the harms and benefits of novel approaches to controlling unwanted animals should be systematically evaluated *before* approval and implementation to determine whether they are genuinely more humane than existing methods (see *Responding to new technologies for Conservation* below). 

### 4.4. First, Do No Harm: Similarities and Differences

So far, I find that, as a Conservation Welfare scientist, I am more similar to, than different from, those working in Compassionate Conservation. The aims and aspirations of the Compassionate Conservation movement and my own reflect a similar attentiveness to the status of the individual wild animal and recognition of some animals’ intrinsic value. As noted above, I would argue that my animal welfare colleagues and I do what we do because we care, because we wish for a better, more compassionate world for animals. However, in some spots, I run into trouble. In particular, I am unsure about how to apply the first Compassionate Conservation tenet in practice. This tenet is: First, do no harm. The ways in which the different aspects of the Compassionate Conservation ethical approach interact to fulfil this tenet, and how they can be reconciled when internal conflict arises is currently unclear [26].

I understand that Compassionate Conservation incorporates a virtue ethic, that a virtuous person is one who makes efforts to avoid inflicting ‘intentional and unwarranted suffering’ and that the focus is on proper conduct, rather than the outcomes of that conduct [5]. But I struggle to reconcile myself to the real possibilities that the best intentioned decision to act or not to act, while virtuous by definition, could have dire consequences for the animals if not well understood [6,28]. Followed exclusively, there is a risk of anthropocentrism if the human virtue and its enactment become more important than the outcomes for the animal itself. 

However, in some descriptions, Compassionate Conservation also appears to incorporate an element of consequentialism [22]. For example, several websites relating to the Centre for Compassionate Conservation (University of Technology Sydney) articulate the movement’s desire to ‘find new ways to conserve and protect species and ecosystems that have *less* impact on the welfare of individual animals’ and to find solutions that *minimize* harms to wildlife (my emphases here) [29,33]. This use of such wording suggests that the ‘right’ conservation decisions or actions are those predicted to have better consequences for individual animals (i.e., less impact on their welfare) whilst achieving conservation goals. How this aligns with the dictum to do *no* harm [5] is unclear. 

Scrutiny before action is also a fundamental first step of the systematic approach to wild animal management advocated by Conservation Welfare [10,17,35]. Decisions about acting or not acting are theoretically based on a weighing-up of the potential harms (to animal welfare and other parties) and benefits (usually to people, directly or indirectly). In reality, it is clear that the need for intervention is often assumed without careful examination [94], and this is something that needs to change, especially as societal views of non-human animals and acceptable treatment change. As noted above, interventions, particularly those that cause intentional ‘harm’ to individual animals, are often undertaken because it is assumed that failing to act will have dire consequences for protecting other aspects of nature, e.g., rare or native animals or historical species assemblages, but such assumptions are increasingly challenged [82,84,95,96]. 

Compassionate Conservationists also favour non-invasive and non-lethal strategies for achieving conservation goals. However, the assumption that these approaches invariably cause less harm to individual animals than invasive or lethal ones should be subjected to careful scrutiny [85,86,87]. Non-invasive methods can sometimes cause significant harms and, in some situations, deciding not to act can cause more harm than acting. For example, restriction of animals’ natural range because of human land use may limit food resources, leading to prolonged hunger and even death from starvation if some form of intervention is not undertaken [22,28,43]. Prospective evaluation of harms and benefits to interested parties, evaluations which may be informed by data gleaned from research and observation via disciplines such as conservation and animal welfare sciences [34], may inform decisions about whether to intervene.

Inevitably, when the possibility of intervening is explored, questions arise: What harms are likely and how can we know? More fundamentally: What is a harm? It seems to me that this is where one of the key differences between Compassionate Conservation and Conservation Welfare lies and one that may not be reconcilable. In contrast to many Compassionate Conservationists [5,16,25,26,31], many animal welfarists contend that death itself is not a harm (however, compare to [97]) and that sometimes a ‘humane death’ can be preferable to a life of intractable suffering or to an imminent ‘inhumane death’ [28,98,99,100]. For instance, might it be better for some stranded whales to be euthanized rather than refloated, if their chances of short-term survival are low and their natural death very unpleasant? In such situations, can killing not be considered an act of compassion [76]? A categorical ‘no’ to this question seems to support the assertion of some conservation biologists that Compassionate Conservation incorporates an animal rights ethic [22].

In the context of death or killing, a common Conservation Welfare perspective is that ‘harms’ can only be experienced by sentient animals up to the point that consciousness is irreversibly lost [99]. This general position reflects the concept that harms are the unpleasant feelings or mental states that arise due to both physical (e.g., injury, disease, inadequate food or water, inappropriate physical or sensory environment) and psychological (e.g., isolation or inappropriate social environment, threat, limited opportunities to express normal foraging or escape behaviours) impacts on the animal [8,101]. Examples of unpleasant mental experiences include pain or discomfort, thirst, hunger, fear, loneliness, boredom, frustration, grief and others [99], some of which are currently poorly understood. Intense, prolonged and/or intractable experiences of these unpleasant states are often characterized as ‘distress’ or ‘suffering’ [102,103], although there are other, more physical definitions [48]. Even in the absence of such negative experiences, a lack of positive experiences such as feelings of security, control, excitement, joy, companionship and others can lead to poor welfare [104]. 

When consciousness is absent (e.g., has not yet begun at a certain point of development) or has been irreversibly lost, we generally contend that such mental states do not arise and thus no harms are inflicted, at least to the ‘target’ animal. Of course, unpleasant mental experiences may arise for other animals as a result of intervention (or no intervention) upon the target animal. Examples might include fear, hunger, thirst or loneliness for dependent or companion conspecifics, or, as has been suggested for both wild and domestic animals, feelings associated with witnessing injury, debility or death in a conspecific [5,105,106,107]; if supported rather than just assumed, such collateral impacts should also be included in harm-benefit analyses when deciding whether to intervene [45]. According to this perspective, the potential for harms should be assessed for all sentient animals whenever they are capable of experiencing them.

It is important to acknowledge that following this approach (i.e., that unconscious animals cannot experience harms) can sometimes cause discomfort or even suffering amongst those people managing wild animals. For example, despite current scientific understanding that fetal mammals are not conscious before birth and are thus not capable of experiencing harms during that stage of development, they do make physical responses to disturbance in utero [108,109]. Similarly, unconscious animals can still show some behavioural and physiological responses after stunning [110,111]. Those responsible for, or witnessing, invasive or aesthetically unpleasant acts on such animals, that *would* cause harm if the animal was conscious, may still feel that these acts are inconsistent with their desire to be compassionate, and they may suffer accordingly. However, while it important to acknowledge these human feelings, care must be taken not to allow human ‘interests’ to dominate in situations where failing to act would cause more harm to the animal than acting.

Regarding questions about the harms that are likely and how we can know, these are precisely the kinds of questions animal welfare scientists attempt to answer, mostly using scientific methods, although it is acknowledged that this is only one way to understand the world and create knowledge, cf. for example religious or indigenous knowledge systems [112,113]. In particular, expanding our understanding of animals’ experiences, both positive and negative, is one of the key foci of animal welfare science [114,115], as well as of researchers in other disciplines [20,116]. To facilitate thoughtful, evidence-based conservation action, it is important to systematically evaluate both the situations likely to generate unpleasant experiences (and/or impair animals’ ability to have positive ones) and whether wild animals can have such experiences at all. Likewise, there is a need to explore ways to modify or refine conservation activities to circumvent or mitigate harms to wild individuals [71,72,117]. 

Sometimes, this type of evaluation causes harm to some individual animals [44,53,87,118,119] and is thus unacceptable to those for whom such intentional harm cannot be justified for any benefit. Compassionate Conservation scientists are clear on this point: priority should be put on developing and applying non-invasive and non-lethal strategies that support conservation goals. In contrast, many Conservation Welfare scientists accept some such harm if the outcomes facilitate convincing arguments for the need to protect animals in the future from situations that cause those unpleasant experiences. As an example, collation of historical research on the ability of non-human mammals to experience unpleasant breathlessness reviewed by [115]) now allows us to strongly advocate against the use of stunning methods that cause such experiences [92,93]. Animal welfare scientists and others also work towards minimizing the harms experienced by animals subjected to such research [45,120,121] and we should continue to do so and to push for stronger requirements to safeguard the welfare of research animals [44]. 

Beyond the notion that killing animals causes them harm, it is not entirely clear what Compassionate Conservationists consider harms to be, how we can know when harms have been inflicted and which non-lethal interventions cause more harm than others. Impacts on individual animals are variously referred to as ‘death and harm…encompassing suffering experienced by individuals and associated costs to social units and populations’ [16], as ‘death and pain’ and as including ‘acute stress and injury’ [25]. Specific examples of harm or suffering include slow, painful death, severe pain before death and dying of dehydration, starvation or exposure [25], but nowhere in the published literature on Compassionate Conservation has a clear characterization of harm or suffering and their recognition been presented.

A further challenge arises when acting or not acting will inevitably have negative impacts on some sentient animal. If we follow the Compassionate Conservation principle of inclusivity, how can we make decisions about whether to act or not to act, if both choices lead to harm to wild individuals [9,26]? A commonly cited example is the choice between controlling or not controlling introduced predators; the former harming the predators and the latter allowing harm to come to native prey [6]. Other examples include leaving wild animals in environments made resource-poor due to human land use, rather than managing population numbers or translocating some individuals to places with more resources [28,122]; preventing wild animals from accessing food resources in their traditional range, including livestock [123]; and exposing captive-bred individuals to live predators before release to improve their post-release survival [124]. In the absence of additional guidance, it is not clear how compassion can be enacted in such complex conservation situations [26]. 

## 5. Responding to New Technologies for Conservation

As discussed above, in order to avoid doing harm to individual wild animals, we must understand and be able to compare, at least qualitatively, the harms likely to arise in different situations [8]. Hypothetical scenarios are useful for exploring virtues and values and revealing implicit ethical beliefs and their practical implications, particularly in complex situations [26]. Here, an emerging scenario, the use of genetic technologies for control of introduced animals, is used to explore the ways each discipline might respond to novel threats to wild animal well-being.

Genetic technologies, specifically ‘gene drives’ and related techniques, are proposed to offer, in the near future, an effective, environmentally friendly and humane method of eradicating populations of unwanted animals for conservation purposes [125,126]:

“…genetic population suppression would be extremely useful for conservation and, unlike traps and poisons, would not cause any animals to suffer.” (Esvelt and Gemmel, 2017 p2) [127].

Gene drives work by promoting inheritance of a particular genomic change to increase its frequency within a population. In the context of pest control, useful genomic changes are those that result in infertility, population level sex biases or even death of individuals at some stage of their lifecycle [126,127,128], ultimately leading to population collapse. However, the humaneness of these genetic changes, is at present assumed, but not assured. There is undeniable potential to improve overall humaneness by eliminating the risk of unintended negative impacts on non-target animals, usually the objects of conservation protection. For example, using genetic techniques specific to the target species would reduce the need for traps and broad spectrum poisons that can cause accidental death or injury to native animals [129]. 

But the dangers of assuming predictable outcomes of genetic modifications are illustrated by the common occurrence, in (highly specific) knockout mice, of phenotypes that were unanticipated or that manifested only under certain environmental conditions [130], as well as unexpected epigenetic effects observed in farm animal transgenesis [131,132,133]. Likewise, naturally occurring human infertility due to various genetic syndromes is sometimes (but not always; [126]) accompanied by multiple co-morbidities that may impact on individual health and welfare [134]. To illustrate, gene drive experts discussing production of infertile mice by inserting a portion of the male *Sry* gene into female embryos concede that it is not yet known how the health and fitness of naturally male ‘carrier’ mice will be affected by carrying two functional copies of the gene [135].

While real concern has been expressed about the ecological safety of genetic technologies for conservation [127,136,137,138] and about different cultural perspectives on altering the ‘essence’ of parts of the natural world [138,139], thus far, I have seen only very broad and passing mention of the potential for harms to the well-being of animals genetically manipulated for the purposes of conservation (Royal Society Te Apārangi 2019 p16) [140]. Importantly, harmful effects are likely to vary with the specific genomic change created and methods used and the species, population, sex and environment of the animals involved [131], so blanket reassurances about humaneness cannot be made. 

As a Conservation Welfare scientist, I respond to this issue by contemplating how we can integrate systematic and scientific evaluation of harms into formal decision-making processes about how, when and which genetic strategies might be employed [131], and how any harms compare to those associated with existing control methods. We may be able to predict some harms, based on our knowledge of the functions of specific genes, but the complex interactions of genetics and environmental effects mean that unanticipated outcomes are likely. Thus, this kind of evaluation will require laboratory (and eventually controlled field) testing of the effects of various gene drives on the animals, both those directly affected by the genomic change and others in those populations over the generations before the population is eradicated. To illustrate, in some species, there are likely to be significant negative impacts to the well-being of individuals if a strong sex bias is created in the population [141,142].

In the absence of this kind of research, which will undoubtedly cause some harm to some sentient subject animals, how can we know whether these methods are consistent with the principles of Compassionate Conservation? Applied to eradicate introduced vertebrate animals, genetic technologies, like existing ‘pest control’ methods, fail to comply with the tenet of *Peaceful coexistence* and probably with *Inclusivity* too. With regard to the first tenet (Do no harm), how can we tell whether and what harm might be inflicted without exploratory research? 

Would Compassionate Conservation scientists dismiss these approaches out of hand as interventions that demonstrate collectivism and instrumentalism? Or is there scope to explore their potential for achieving widely desired conservation aims whilst also considering the interests of the individual animals, and perhaps offering better outcomes than those associated with methods currently used *en masse* in places like New Zealand (lethal poisons and live traps followed by blunt force trauma; [143])? With regard to the Compassionate Conservation priority on non-invasive and non-lethal conservation activities, some proposed genetic technologies would have non-lethal outcomes and, after initially engineering the modification into embryonic animals, the genomic changes would be propagated throughout the wild population with no further intervention from humans. These technologies may therefore be viewed as relatively non-invasive. However, as discussed above, it is possible that suffering will be experienced by some individuals of some species with some intentionally engineered non-lethal mutations. Accordingly, I am uncertain how the Compassionate Conservation tenets would guide decision-making about whether these novel tools qualify as compassionate or not.

Regardless of these uncertainties, these sorts of techniques are likely to be on their way. As noted by Esvelt and Gemmel (Esvelt and Gemmel 2017 p5) [127]:

“Even if… [emerging gene drive technologies] don’t work well enough, interest from the Defense Advanced Research Projects Agency (DARPA) Safe Gene program and other funders makes it likely that a superior strategy will be invented soon.”

Unevaluated, there is potential for these technologies to inflict significant harm to enormous numbers of individual sentient animals once applied on a large scale. My preference, as a Conservation Welfare consequentialist, would be to systematically explore those harms and use that knowledge to decide whether or not implementing those technologies is ethically justifiable and/or how any harms can be minimized. A great deal of money and political influence is driving countries like New Zealand ever closer to the application of these technologies and I worry that calling on compassion will not be enough to stop the momentum even if harms to animals are likely [82,144]. We know too well that the absence of evidence is readily taken to be evidence of absence; without scientific evidence, the public may easily be convinced that genetic technologies are, as has been implied, more humane than existing approaches. 

This means working together across various disciplines (e.g., genetics, conservation, animal behaviour, ecology, animal welfare, environmental science, bioethics, social policy) to prepare and integrate methods for evaluating the animal welfare impacts of genetic technologies into the official approval processes controlling their development and use. These reflections and conversations about the broad ethical implications of new technologies applied to animals should take place among collaborators and stakeholders at the earliest stages of planning [26]. Importantly, this means putting animal welfare front and centre in discussions of the ethics and practicalities of implementing genetic technologies for conservation, explicitly requiring independent, validated, science-based and systematic animal welfare assessments by those with appropriate expertise as part of applications for approval to conduct research to further develop these technologies, and as part of what may become ‘routine’ environmental approvals to release engineered individuals in field trials or *en masse*. For the reasons outlined above, Conservation Welfare appears to offer the clarity and structure required to support this process. 

## 6. Discussion

Some argue that all ethics can do is eliminate confusion and clarify the issues, i.e., what is at stake. In the present case, although both Compassionate Conservation and Conservation Welfare proponents are focused on consideration of the well-being of individual wild animals subject to conservation decisions, the ethical foundations are fundamentally different. This means that what is considered the right or good thing to do will often differ, with Conservation Welfarists being focused on the overall outcomes of decisions (for animals and humans) and Compassionate Conservationists on some aggregate of the intent of the action, the outcomes for the animals and the avoidance of killing. Neither approach is faultless or sufficient to address the multiple concerns people hold about conservation of nature.

Conservation Welfare (as a manifestation of the animal welfare ethic) is problematic in that it will permit large numbers of sentient animals to suffer greatly as long as the greatest overall good is achieved (which usually cannot be predicted or evidenced [8]). In addition, although ostensibly underpinned by a utilitarian ethic, in practice, this is not followed faithfully. In reality, the human purpose for ‘using’ the animal plays a key role in decision-making. Animal uses that are perceived to be ‘necessary’ are permitted regardless of the degree of suffering. Luckily, what is considered ‘necessary’ is not fixed, allowing for progressive retractions of permission to cause harm as social views on acceptable treatment of animals changes [50]. A related limitation of the practical application of the animal welfare ethic is that the sentience threshold for moral considerability is often violated; different acts are permitted on the same species of animal, with the same capacity for suffering, when the acts have different purposes [50,56]. Examples include permission to cause harm in research that would not be permitted for a companion animal of the same species or when animals are labelled as pests in some locations and thus exempted from welfare protection. 

Acknowledging that no single foundational principle is likely to satisfy concerns that encompass multiple levels of biological organization (individuals, populations, ecosystems, biodiversity), Fraser [8] proposed a practical ethic for animals. This approach is consistent with elements of both Compassionate Conservation and Conservation Welfare and reflects the pragmatic approach often taken in real-life conservation-welfare conflicts. It is founded on ‘mid-level’ principles designed to be responsive to people’s actual concerns about current problems in the real world and to offer a framework for decision-making that satisfies people’s multiple simultaneously held values, e.g., concern for the welfare of individual animals and for safeguarding biodiversity. These principles are: 1. Provide animals with good lives in our care; 2. Treat suffering with compassion; 3. Be mindful of unseen harm; 4. Protect life-sustaining processes and balances of nature. 

Some Compassionate Conservationists appear to interpret Fraser’s second principle (Treat suffering with compassion) as an absolute (i.e., First, do no harm), and a number of recent publications in conservation biology journals have been critical of a perceived oversimplification of the issues at stake [22,26,28,76]. However, Fraser was explicit that the compassion principle does not call for the elimination of all animal suffering, rather that compassion should be applied in relevant contexts and with consideration of the other principles. Compassionate Conservation has also been criticized for focusing on intentionally inflicted (i.e., direct) harms and ignoring predictable though indirect or ‘unseen’ harms [28]. In contrast, it could be argued that the Animal Welfare ethic fails to uphold Fraser’s second principle because it de-prioritizes compassion when benefits to humans are seen to outweigh ‘necessary’ harms to animals [16,50].

Fraser’s framework also acknowledges that the harms and benefits caused by human activities, including conservation, vary greatly depending on how they are carried out. So to dismiss categories of activities as inherently compassionate or un-compassionate without regard for the context-dependent outcomes for animals and people is unlikely to be acceptable to many [76]. Some critics have gone so far as to claim that this categorical approach means that Compassionate Conservation is likely to achieve neither the goal of alleviating suffering (compassion) nor protection of life-sustaining processes and balances of nature (conservation) [22]. 

Another major criticism of Compassionate Conservation, as it is currently articulated, is its failure to provide guidance on resolving real-life conflicts [7,9]. In particular, Rohwer and Marris [26] encourage Compassion Conservationists to be more explicit about what a virtuous conservationist would do in complex situations, such as when harm will be done by humans regardless of the decision taken or when the goals of maintaining biodiversity and avoiding suffering cannot be reconciled. While Wallach et al. offer suffering alongside those animals that are harmed as the appropriate course of action for Compassionate Conservationists, this does not progress our understanding of, or ability to avoid or reduce, such suffering in the future. This lack of clarity poses a real risk to the advancement of individual wild animal welfare in conservation decision-making [6,27,76,145] and highlights, for me, the value of differentiating the more permissive (though imperfect) Conservation Welfare approach. 

When conflict arises among his mid-level principles, Fraser [8] delineates features for decision-making: Considering the number of animals affected, the degree and duration of the effects on individuals (harms and benefits), whether the action will have long-lasting or irreversible effects (e.g., extirpation or extinction) and the degree to which people can control or mitigate harms or provide benefits. The first two features can be informed by the application of ecology and animal welfare science methods and the latter two by approaches such as ecological modelling, adaptive management and explorations of human motivations and behaviours using social sciences methods. The focus is on minimizing animal suffering to the greatest extent possible (particularly suffering that could be avoided) rather than eradicating it at the cost of achieving conservation goals. This approach is consistent with the principles of Conservation Welfare and the goal now is to have these principles applied more broadly in conservation decision-making and practice.

Ultimately, individuals who care about wild animal welfare must come to their own conclusions about appropriate ethical principles to follow. However, at higher levels of decision-making, ethical frameworks are needed to protect the multiple aspects of nature for which many in society have concern. There is a real and growing risk that the strong focus of Compassionate Conservationists on avoiding killing animals and on enacting compassion even in the face of evidence that the outcomes will be poor for individual animals and/or for achieving conservation goals will hinder attempts to integrate consideration of individual animal well-being into mainstream conservation decision-making, policy and practice [6,22,27,28,76]. 

In contrast, there is already recognition of the value of the principles of the Animal Welfare ethic and supporting scientific approaches (Conservation Welfare) in conservation [2,17,28,40,45,46,52,85,87,91,146,147,148]. Conflating Conservation Welfare and Compassionate Conservation is already confusing many and is likely to cause wholesale rejection of individual animal welfare as a key factor to consider in modern conservation decision-making [6,22,27]. Despite its limitations, I propose that Conservation Welfare offers a more practical and palatable approach to systematically integrating consideration of individual wild animal welfare. In large measure this is because it is clear about which animals are to be considered (usually those defined as ‘animals’ in the relevant country’s legislation), what harm/suffering are and how they can be assessed, and how to fulfil our obligations to consider animal welfare as part of contextualized decision-making, i.e., by minimizing harms whenever possible. A challenge going forward will be negotiating which conservation ‘uses’ of animals are ‘necessary’ and thus, ethically permissible, and I welcome open and informed debate among conservationists, Animal Welfarists, Compassionate Conservationists and communities on this important subject.

## 7. Conclusions

Compassionate Conservation and Conservation Welfare are two disciplines whose practitioners advocate consideration of individual wild animals within conservation practice and policy. However, they are not, as is sometimes suggested, synonymous. Compassionate Conservation and Conservation Welfare are based on different underpinning ethics, which lead to different approaches to the evolution of the traditional conservation paradigm and its associated activities. Conservation Welfare scientists are seeking to engage with conservation scientists with the aim of facilitating ongoing incremental improvements in all aspects of conservation policy and practice. In particular, we call for more consistent and rigorous consideration of ‘pest animal’ welfare when evaluating the continued use of existing control methods and the development of novel methods. In all cases, animal welfare impacts should be purposefully, scientifically and systematically investigated by those with appropriate expertise rather than assuming that such impacts will not occur, as often happens, in part due to the failure to include such experts in research and policy design teams. 

Currently, there is a lack of clarity about how the tenets of Compassionate Conservation can be interpreted and applied by conservation practitioners to improve outcomes for individual wild animals in the broader range of situations. This may hinder uptake by traditional conservationists of the approaches advocated by champions of Compassionate Conservation. In particular, clarification is needed about the ways in which ‘Do no harm’ can be enacted in apparently intractable situations where both acting and failing to act will result in harm to individual wild animals. Importantly, following the Compassionate Conservation approach, the wild animals that can experience harms, the kinds of harms they can experience and how we can know about and confidently evidence those harms is currently uncertain. In this regard, Conservation Welfare appears to provide an advantage over Compassionate Conservation for assessing the ways in which novel conservation activities might represent improvements in animal welfare outcomes. For these reasons, Conservation Welfare and allied scientific methods (i.e., animal welfare science) may offer modern conservationists a more palatable approach to integrating evidence-based consideration of individual sentient animals into conservation practice and policy.
